# Effect of Swirling Flow Nozzle on Fluid Flow and Solidification in a Round Bloom Continuous Casting Mold

**DOI:** 10.3390/ma15238474

**Published:** 2022-11-28

**Authors:** Jianli Wang, Jiayu Zhu, Yongkun Yang, Weian Wang, Guoxing Qiu, Xiaoming Li

**Affiliations:** School of Metallurgical Engineering, Xi’an University of Architecture and Technology, Xi’an 710055, China

**Keywords:** round bloom, numerical simulation, fluid flow, solidification, swirling flow nozzle, P91 steel, mold curvature

## Abstract

The nozzle structure has an important effect on the fluid flow in the mold, which can significantly improve the solidified shell and product quality of alloy steel round bloom. The transient fluid flow, heat transfer, and solidification behavior under different nozzle structures and mold electromagnetic stirring (M-EMS) are investigated using a 3D transient mathematical model. The results show that a third small recirculation zone appears near the meniscus after the application of the swirling flow nozzle (SFN). The impact depth of SFN is shallower than that of the original submerged entry nozzle (SEN) impact, and the lower circulation zone is shifted upward. The horizontal swirling flow generated by SFN can significantly weaken the washing of the initial shell by high-temperature steel and improve the uneven growth phenomenon of the inner and outer curved solidified shell caused by mold curvature. The swirling flow produced by M-EMS in the mold can also improve the washing of the initial shell by the high-temperature jet and the uneven growth of the inner and outer curved shell. M-EMS can expand the high-temperature zone in the upper part of the mold, promote the superheat dissipation of the molten steel, and promote the growth of the solidified shell. In addition, after the application of M-EMS, the tangential velocity of –15° SFN in the meniscus is smaller, and the resulting liquid level fluctuation is lower at 5.07 mm, which is less likely to produce slag entrapment and is conducive to improving the quality of round bloom.

## 1. Introduction

Continuous casting round bloom of alloy steel is widely used in high-pressure boiler tubes, high-speed rail axles and wheels, wind power flanges, and other fields. As the section of the round bloom increases, the solidification rate decreases significantly, and the flow field distribution in the mold is different from that in the small cross section. Reasonable control of molten steel flow in round bloom mold can effectively reduce both internal and surface defects [1,2]. The main measures to improve the mold flow are changing the nozzle structure and applying mold electromagnetic stirring (M-EMS).

The molten steel enters the mold through a submerged entry nozzle (SEN) for solidification. The flow behavior of molten steel at different process conditions was studied by optimizing nozzle parameters (port angle, port size, immersion depth, port shape), casting speed, nozzle blowing, etc. [3]. It provides an important reference for improving the flow field distribution of molten steel in the mold. The swirling-flow blade SEN improves the steel flow in SEN by installing swirl blades in SEN [4]. This nozzle can effectively reduce the steel impact depth in the mold, accelerate the steel superheat dissipation, and reduce the effect of the jet on the initial billet shell [5]. Plant trials by Yokoya et al. showed that the swirling-flow blade SEN reduced pinhole defects and improved the surface quality of steel sheets [6,7]. However, the energy efficiency of the blade was only 20%, resulting in limited swirl intensity [7]. Swirl blades were scoured by high-temperature steel flow and inclusions deposited, limiting their long-term use in production.

Electromagnetic swirling flow in the nozzle (EMSFN) by installing an electromagnetic stirring device outside the SEN uses electromagnetic force to create a rotating flow of steel in the nozzle. The swirling flow intensity can be adjusted with different parameters of the EMSFN [8,9]. Wu et al. [10,11] used EMSFN together with M-EMS for industrial trials and found that when the stirring directions of the two electromagnetic devices were opposite, the level fluctuation of the free surface was reduced, and the equiaxed crystal ratio was increased by 15%. However, the cost of installing and maintaining electromagnetic devices is high, and the limited space between mold and tundish limits the further development of EMSFN.

Recently, a swirling flow tundish SEN design was proposed, where a tube-shaped swirling flow generator was installed in a conventional tundish [12]. Ni et al. found that this design achieved the same effect as a swirling flow nozzle by establishing a 3D mathematical model and physical [13,14]. However, the structure of this specially designed tundish is different from that of the traditional tundish, so it is necessary for the steel plants to completely transform the original tundish.

Sun [15] and Lin et al. [16] found that a horizontal swirling flow could be produced when using a swirling flow nozzle (SFN) with four tangential outlet walls. Compared with the normal nozzle, it is found that the SFN tangential outlet design can change the vertical jet into a horizontal swirl flow, promote the superheat dissipation of molten steel, and improve the defects such as central porosity and center crack of round bloom [17]. However, the swirling flow intensity generated by SFN is low, and the high-temperature molten steel from the nozzle is less uniformly distributed. Therefore, it is often necessary to use it together with M-EMS technology.

M-EMS improves the flow field and temperature distribution of molten steel in the mold by the non-contact stirring of molten steel by electromagnetic force [10,11,18]. The forced convection caused by electromagnetic stirring is beneficial to the formation and floating of the free nucleus to obtain a higher equiaxed grain rate, reduce the defects such as central segregation of the billet [19], and improve the quality of the billet [20,21]. Li et al. [22] reported that the flow field, temperature, and solute distribution were symmetric in a vertical mold and non-symmetrical in a curved mold.

The above studies showed that swirling flow nozzle structure and M-EMS can effectively improve the flow of molten steel in the mold and improve the billet quality. In past studies, when studying the swirling flow nozzle, researchers have ignored mold curvature. However, mold curvature inevitably affects the steel flow in the mold, making the flow field in the mold more complex. In this study, a three-dimensional mathematical model in the curve mold was established, the effects of the original SEN and SFN on the flow field, temperature field, and shell thickness were compared and analyzed, and the influences of different stirring directions of SFN and M-EMS on the steel flow in the mold were discussed, which provides a new idea for improving the solidification quality of the round bloom.

## 2. Model Descriptions

### 2.1. Basic Assumptions

In the actual production of continuous casting round bloom, the site conditions were complex. In order to reduce the complexity of the model and improve its computational efficiency, the following assumptions were made:

(1) The high-temperature molten steel was assumed to be an isotropic and Newtonian incompressible fluid, and the viscosity, specific heat capacity, and thermal conductivity were assumed to be constant;
(2) The effects of the shrinkage of the round bloom and the oscillation of the mold on the flow of molten steel were not considered;
(3) The local solid–liquid interface at the solidification front was assumed to be in local thermodynamic equilibrium during the computational process;
(4) A simplified electromagnetic stirrer structure was adopted, and air was used instead of cooling water, stainless-steel protective sleeve, and insulating material in the electromagnetic stirrer;
(5) The influence of the Joule heating caused by the induced current was ignored in the heat transfer calculation.

### 2.2. Governing Equations

(A) Electromagnetic field model

Under low-frequency conditions, the electromagnetic field can be calculated by solving Maxwell’s equations:

(1) $$\nabla \times E=-\frac{\partial B}{\partial t}$$

(2) ∇×H=J

(3) ∇⋅B=0

(4) J=σ⋅E

where *E* is the electric field intensity, V/m; *B* is the magnetic flux density, T; *H* is the magnetic field intensity, A/m; *J* is the current density, A/m^2^; *σ* is the electric conductivity, S/m, and *t* is time, seconds. The time-averaged Lorentz force is calculated using the following equation:

(5) $$F_{\mathrm{mag}}=\frac{1}{2}Re\left(J\times B\right)$$

where *Re* is the real part of a complex number, and *F*_mag_ is the time-averaged Lorentz force.

(B) Fluid flow model

The fluid flow model is mainly based on the continuity and the momentum equations:

(6) $$\frac{\partial \rho }{\partial t}+\nabla \cdot (\rho u)=0$$

(7) $$\frac{\partial }{\partial t}(\rho u)+\nabla \cdot (\rho uu)=-\nabla p+\nabla \cdot [\mu_{\mathrm{eff}}(\nabla u+\nabla u^{T})]+\rho g+\rho g\beta_{T}\left(T-T_{\mathrm{ref}}\right)+S_{m}+F_{\mathrm{mag}}$$

where *ρ* is the density, kg/m^3^; *u* is the flow velocity, m/s; *p* is the pressure, Pa; g is the gravitational acceleration, m/s^2^; *β*_T_ is the thermal expansion coefficient, K^−1^; *T* is the temperature, K; and *T*_ref_ is the reference temperature, K, which is set as liquidus temperature.

S_m_ represents the momentum sink due to the reduced porosity in the mushy zone described by Darcy’s law:

(8) $$S_{m}=\frac{{\left(1-f_{1}\right)}^{2}}{\left(f_{l}^{3}+\xi \right)}A_{\mathrm{mush}}\left(u-u_{s}\right)$$

where *f*_l_ is the liquid fraction; *ξ* is a very small positive number; A_mush_ is the mushy zone constant; *u*_s_ is the velocity of solid phase, m/s; and *μ*_eff_ is the effective viscosity of turbulence flow, Pa·s, calculated as follows:

(9) $$\mu_{\mathrm{eff}}=\mu_{l}+\mu_{t}=\mu_{l}+\rho C_{\mu }\frac{k^{2}}{\varepsilon }$$

where *μ_l_* and *μ_t_* are the laminar viscosity and turbulent viscosity, respectively, kg m^−1^ s^−1^; *C_μ_* = 0.09; *k* is the turbulent kinetic energy, m^2^/s^2^; and *ε* is turbulent dissipation rate, m^2^/s^3^.

The turbulence was governed by the realizable *k-ε* two-equation model. K and ε were solved using the following equations:

(10) $$\frac{\partial }{\partial t}(\rho k)+\nabla \cdot (\rho ku)=\nabla \cdot [(\mu_{l}+\frac{\mu_{t}}{\sigma_{k}})\nabla k]+G_{k}+G_{b}-\rho \varepsilon +S_{k}$$

(11) $$\frac{\partial }{\partial t}(\rho \varepsilon )+\nabla \cdot (\rho \varepsilon u)=\nabla \cdot [(\mu_{l}+\frac{\mu_{t}}{\sigma_{\varepsilon }})\nabla \varepsilon ]+C_{1\varepsilon }\frac{\varepsilon }{k}\left(G_{k}+C_{3\varepsilon }G_{b}\right)-C_{2\varepsilon }\rho \frac{\varepsilon^{2}}{k}+S_{\varepsilon }$$

where *G_k_* is the generation of turbulence kinetic energy due to the mean velocity gradients; *G_b_* is the generation of turbulence kinetic energy due to buoyancy; *C_1_ε*, *C_2_ε*, and *C_3_ε* are empirical constants; and *σ_k_* and *σ_ε_* are the turbulent Prandtl numbers for *k* and *ε*, respectively. The recommended values for *C_1_ε*, *C_2_ε*, *C_3_ε*, *σ_k_* and *σ_ε_* are 1.44, 1.92, −0.33, 1.0, and 1.3, respectively. *S_k_* and *S_ε_* are source terms.

(C) Heat transfer and solidification model

The energy conservation equation was solved to study the temperature field in the continuous casting process:

(12) $$\frac{\partial }{\partial t}(\rho H)+\nabla \cdot (\rho uH)=\nabla \cdot (k_{eff}\nabla T)$$

(13) $$H=h+\Delta H=h_{ref}+\int_{T_{ref}}^{T}C_{p}dT+f_{l}L$$

where *H* is the total enthalpy, J/kg; *T* is the temperature, K; *h_ref_* is the reference enthalpy, J/kg; *T_ref_* is the reference temperature, K; *C_p_* is the specific heat, J/(kg·K); *L* is the latent heat, J/kg; and *k_eff_* is the effective thermal conductivity.

### 2.3. Boundary Conditions

By considering the adiabatic effect of protective slag, the molten steel surface was set to zero shear condition and adiabatic condition. The velocity inlet boundary condition was adopted. The inlet temperature was set to the pouring temperature. The inlet velocity was obtained from Formula (14) based on mass conservation. The turbulent kinetic energy k and the turbulent dissipation rate ε were calculated using the following equations:

(14) $$u_{\mathrm{in}}=\frac{u_{c}\cdot S_{\mathrm{out}}}{S_{\mathrm{in}}}$$

(15) $$k=0.01u_{\mathrm{in}}^{2}$$

(16) $$\varepsilon =\frac{k^{1.5}}{R_{\mathrm{in}}}$$

where *u*_in_ is the velocity-inlet, m/s; *u*_c_ is the casting speed, m/s; *S*_out_ is the area in the cross section of the round bloom, m^2^; *S*_in_ is the area in the cross section of SEN inlet, m^2^; and *R*_in_ is the internal radius of the SEN inlet, m.

The heat transfer boundary condition of the mold was expressed as follows:

(17) $$q_{m}=a-b\sqrt{z/u_{c}}$$

where *q*_m_ is the heat flow density; a is a constant, the size of 2,680,000 W/m^2^; and b is expressed as follows:

(18) $$b=\frac{1.5\left(a-\bar{q}\right)}{\sqrt{L_{m}/u_{c}}}$$

where *L*_m_ is the effective length of the mold, m; and $\bar{q}$ is expressed as follows:

(19) $$\bar{q}=\frac{C_{W}\times Q_{W}\times \Delta T}{S_{\mathrm{eff}}}$$

where *C*_W_ is the cooling water specific heat capacity, J/(kg·K); Q_w_ is the mold cooling water flow rate, kg/s; ∆*T* is the mold cooling water temperature difference, K; and S_eff_ is mold effective area, m^2^.

The heat transfer condition in the secondary cooling zone was determined by (20), and the convective heat transfer coefficient *h_spray_* was calculated by (21).

(20) $$Q_{s}=h_{spray}\left(T_{billet}-T_{spray}\right)$$

(21) $$h_{spray}=0.581W^{0.451}\left(1-0.0075T_{spray}\right)$$

where *h_spray_* is the convective heat transfer coefficient, J/(m^2^·K); *T_billet_* is the round bloom surface temperature, K; *T_spray_* is the cooling water temperature, K; and *W* is the cooling water flow rate, L/min.

At the bottom of the calculation area, the fully developed flow conditions were adopted, and the normal gradients of all variables were set to zero.

### 2.4. Solution Procedure

In order to couple the electromagnetic field with the flow of molten steel, the Maxwell equations were solved by ANSYS Electronics-maxwell 2021 software, and the three-dimensional distribution of the electromagnetic field in continuous casting round bloom was calculated. Subsequently, magnetic field data were transformed into the required format for the Fluent magnetohydro dynamics (MHD) module. The semi-implicit method for the pressure-linked equation (SIMPLE) algorithm was used to simulate the coupling of steel flow, heat transfer, solidification, and electromagnetic stirring. The residual of the energy equation was set to 10^−6^, and the remaining equation residuals were set to 10^−4^. The simulated continuous casting process was considered to reach a steady state when the monitoring points of the steel flow rate and the temperature reached a steady state at the outlet.

**Figure 1:**
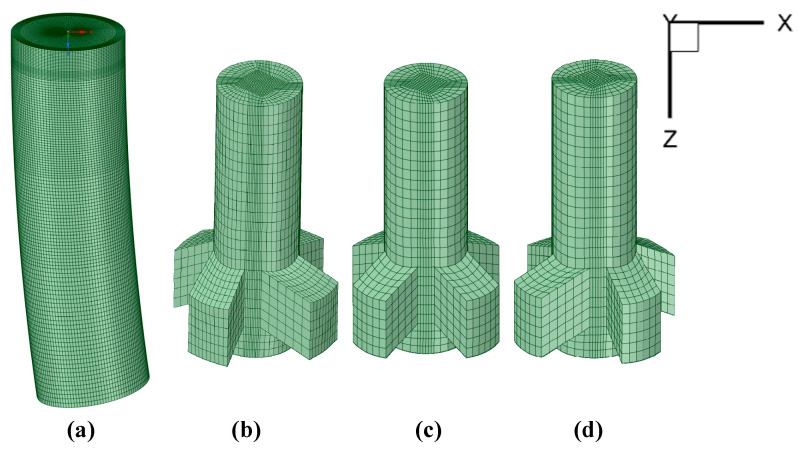
Schematic diagram of the mesh of the round bloom 3D model (**a**) round bloom mesh (**b**) –15° SFN mesh (**c**) 0° SEN mesh (**d**) +15° SFN mesh.

**Figure 2:**
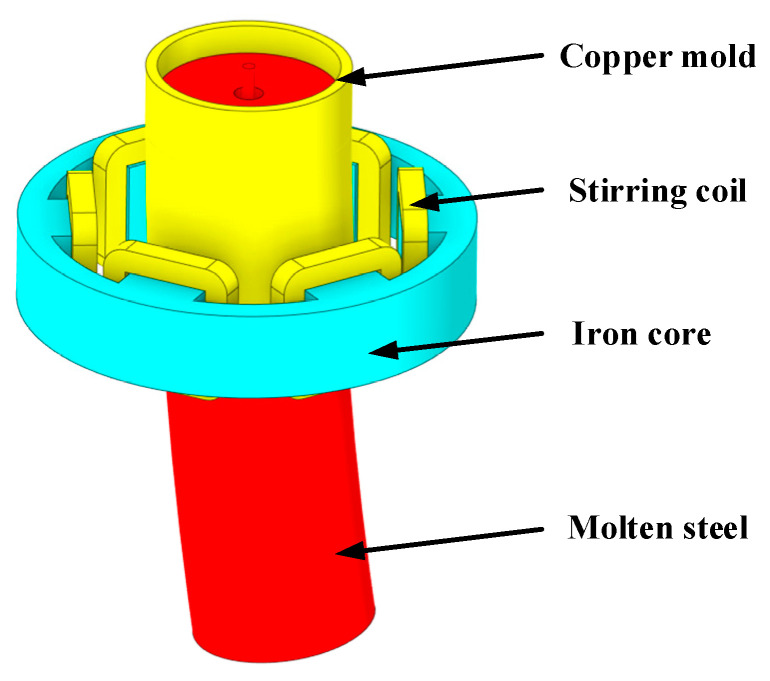
Geometrical model of electromagnetic stirrer.

Figure 1 shows the schematic diagram of the mesh of the round bloom 3D model. The origin of coordinates was located at the center of the meniscus, the *Z*-axis was the direction of gravity, and the total number of meshes was about 690,000. The geometric dimensions of the three kinds of nozzles are shown in Table 1. The geometrical model of the electromagnetic stirrer is shown in Figure 2. Table 2 shows the chemical compositions of the round bloom production steel grade P91. The process conditions and physical parameters used in this study are listed in Table 3. In this paper, in order to better observe the simulation results, the aspect ratio of the display results was adjusted during post-processing. The SFN rotated 15° to the left, the original SEN and the SFN rotated 15° to the right are named –15° SFN, 0° SEN, and +15° SFN, respectively.

## 3. Results and Discussions

### 3.1. Model Validation

(1) Flow field model

**Figure 3:**
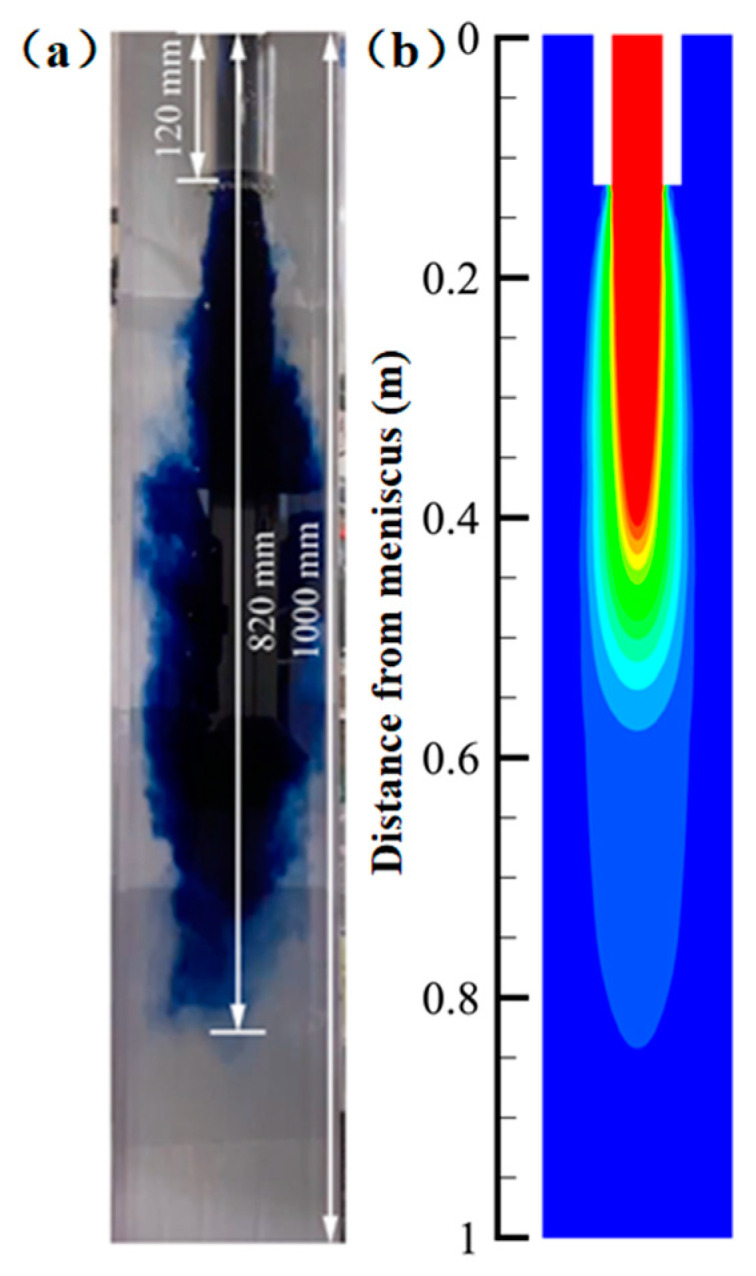
Simulated and water-mode flow field distribution (**a**) water-mode results, (**b**) numerical simulation results.

In order to verify the accuracy of the calculation results, the experimental data provided by Li et al. [23] were brought into the research model, and the predicted numerical simulation results were obtained. Compared with the results of the water model conducted by the authors, as shown in Figure 3, the numerical simulation results were basically consistent with the fluid flow behavior and impact depth simulated by the blue tracer, which indicates that the numerical simulation developed in this paper to analyze the effect of the steel flow field in the mold is feasible.

(2) EMS model

**Figure 4:**
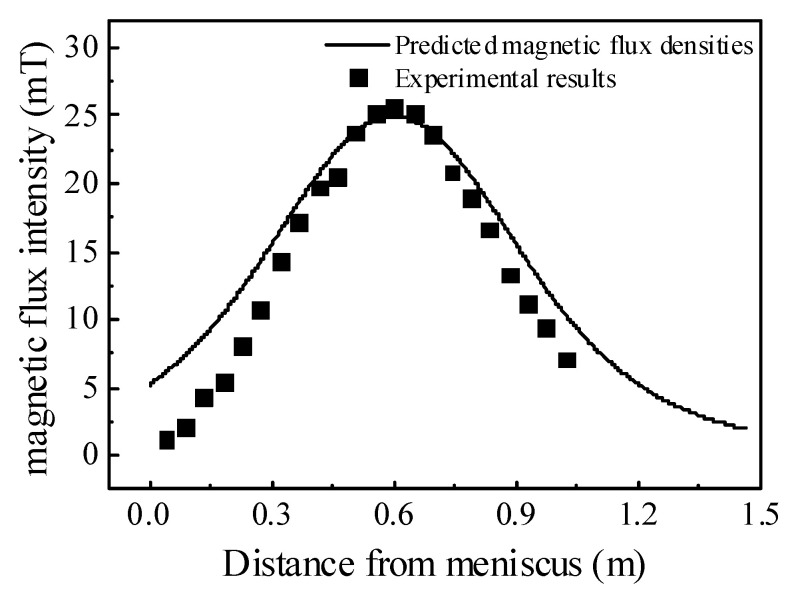
Simulated and measured results of the magnetic flux intensity.

Jauregui and Silva [24] reported that validation using other numerical solutions as a reference model is a technique that compares the results obtained from the other numerical methods that were previously validated. Analogically, Ren et al. [25] conducted industrial measurements for the electromagnetic field of Φ600 large round bloom, which was consistent with the round bloom size in this paper and can be used to validate the electromagnetic model. Figure 4 shows the comparison between the predicted results of the electromagnetic model and the measured results. Based on the assumptions and simplification in electromagnetic field modeling, the predicted results along the casting speed direction of the round bloom were in agreement with the measured data. Therefore, the electromagnetic model was considered reasonable.

(3) Solidification model

**Figure 5:**
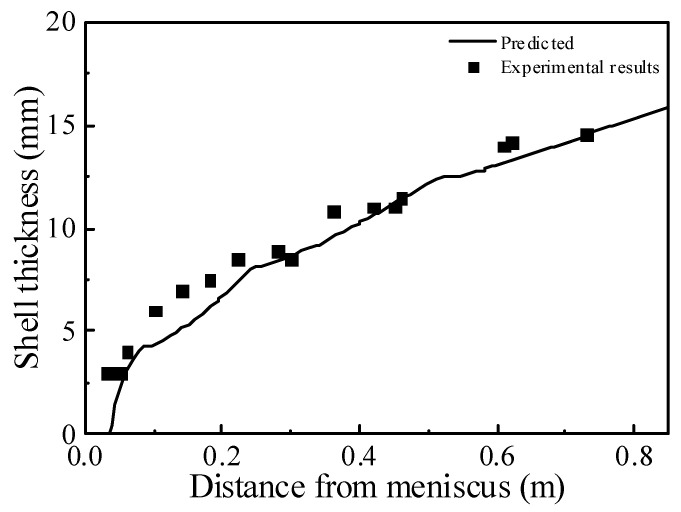
Validation of solidification model.

In order to verify the accuracy of the solidification model, the numerical simulation calculation was conducted according to the slab mold data given by predecessors [26], and the numerical simulation results were compared with the reported solidified shell thickness at the center of the narrow face of the slab mold. As shown in Figure 5, the predicted shell thickness and the experimentally obtained were in good agreement.

### 3.2. Effect of Nozzle Structure on the Steel Flow and Heat Transfer

#### 3.2.1. Effect of Nozzle Structure on the Steel Fluid Flow

**Figure 6:**
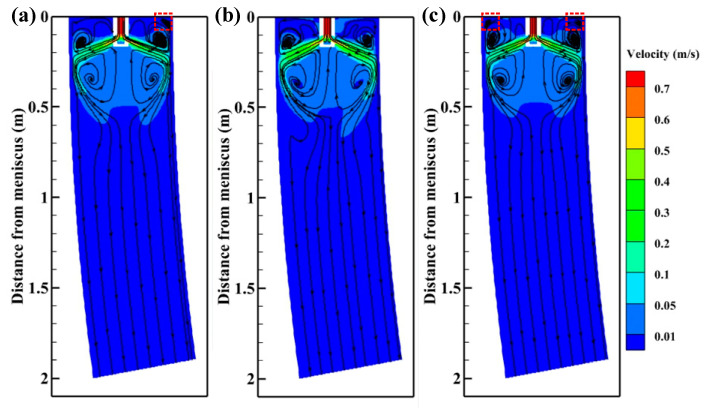
Velocity contours and streamlines on the plane Y = 0 m without M-EMS (**a**) –15° SFN, (**b**) 0° SEN, and (**c**) +15° SFN.

Figure 6 depicts the velocity contours and streamlines on the plane Y = 0 m without M-EMS. The liquid steel enters the mold through the SEN and directly impacts the initially solidified shell, and forms two longitudinal circulations. In the upper circulation, the molten steel moves upward along the solidification front and extends to the meniscus, which can prevent meniscus solidification and promote the melting of mold flux. In the lower circulation, part of the molten steel moves downward along the solidification front, and the other part flows upward to the bottom of the nozzle after reaching the maximum depth. The lower recirculation zone forms a greater range of steel recirculation, which can promote the floatation of inclusions. When SFN is used, a third small recirculating zone is formed near the meniscus, a third small recirculating zone is produced by –15° SFN, and two third small recirculating zones are generated by +15° SFN. The third small recirculating zone is formed by the relative motion between the molten steel flowing from SFN and the molten steel flowing upward. The third small recirculating zone can accelerate the flow of molten steel to the meniscus and provide heat for mold flux. The impact depth of SFN on the Y = 0 m plane is shallower than that of 0° SEN, and the lower circulation zone moves upward. The center of the upper circulation zone generated by 0° SEN is 0.132 m from the meniscus, and the center of the lower circulation zone is 0.366 m from the meniscus. The center of the upper circulation zone produced by +15° SFN is 0.130 m from the meniscus, and the center of the lower circulation zone is 0.357 m from the meniscus. The position of the lower circulation zone moves upward to improve the cooling efficiency of the mold. Compared with the 0° SEN, the remarkable characteristic of SFN is that the molten steel produces a horizontal swirl, which prolongs the residence time of the molten steel in the mold.

**Figure 7:**
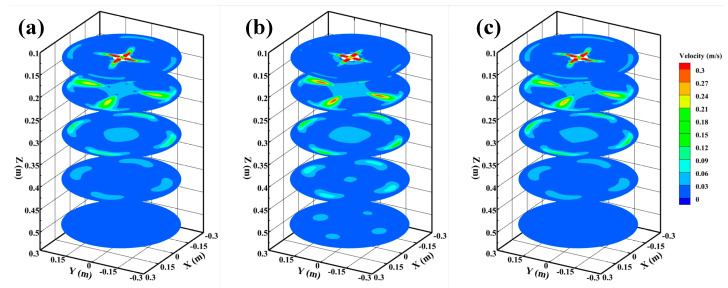
Velocity distributions in cross section of strand without M-EMS (**a**) –15° SFN, (**b**) 0° SEN, and (**c**) +15° SFN.

Figure 7 presents the velocity distributions in cross section of the strand without M-EMS, and the velocity distributions of Z = 0.13 m, Z = 0.2 m, Z = 0.3 m, Z = 0.4 m, and Z = 0.5 m plane flow fields are shown in the figure. On the Z = 0.2 m plane, the molten steel flows out from the nozzle in four high-speed jets. The molten steel of 0° SEN flows out in a linear jet, while the molten steel of SFN flows out with a certain rotation angle. The steel flowing from SFN appears to increase locally near the solidification fronts, and the increase in flow rate corresponds to the third small reflux zone in Figure 6. The jet flow from 0° SEN on the Z = 0.2 m plane is more concentrated and has a higher velocity. When SFN is used, the jet aggregation phenomenon is reduced, and the jet distribution is more uniform. As the steel jet flows and the axial distance increases, momentum exchange occurs between the high-speed jet and the surrounding steel, which makes the jet carry a large amount of low-speed molten steel, resulting in the gradual attenuation of the velocity. Due to the existence of the rotation angle of SFN, the radial velocity range of the jet is greater; the stronger the ability to carry low-velocity steel, the faster the velocity decay and the more uniform velocity distribution. When the steel flows to the Z = 0.3 m and Z = 0.4 m planes, all nozzles have higher flow velocity near the solidification fronts, and the flow velocity distribution of SFN is more uniform. When the molten steel flows to the Z = 0.5 m plane, the velocity distribution of SFN is almost the same, but the velocity of 0° SEN has a local peak value, which also indicates that the impact depth of the 0° SEN jet is deeper.

**Figure 8:**
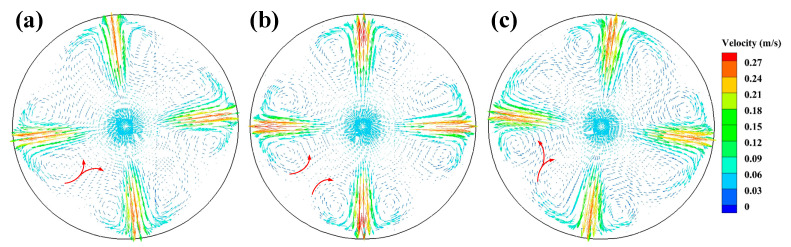
Velocity vector on the plane Z = 0.2 m without M-EMS (**a**) –15° SFN, (**b**) 0° SEN, and (**c**) +15° SFN.

In order to further investigate the reason for the more uniform flow velocity generated by the SFN, the velocity vector distributions of the 0° SEN and ±15° SFN on the Z = 0.2 m plane are given in Figure 8. The steel flowing from the 0° SEN forms four high-speed jets, and two vortexes are formed after each jet impacts the mold wall. When SFN is used, the horizontal swirl occurs after the four jets pass through the tangential rotation angle side port, which weakens the impact of the high-speed jet on the mold wall, and two vortexes of different sizes are formed after impacting the wall. The vortex of the +15° SFN on the right side of the jet flowing is larger. Some of the molten steel on the right side of the vortex flows back to the bundle at the SFN, and the other part flows to the vortex zone adjacent to its side port, making the steel flowing from the SFN more fully mixed. The steel flow of –15° SFN is similar to that of +15° SFN. The horizontal velocity by SFN formation can promote the breakage of the front end of columnar crystals by the steel, forming a large number of dendrite fragments that can be used as nuclei and increasing the probability of equiaxed crystal formation [16].

#### 3.2.2. Effect of Nozzle Structure on Heat Transfer and Solidification

**Figure 9:**
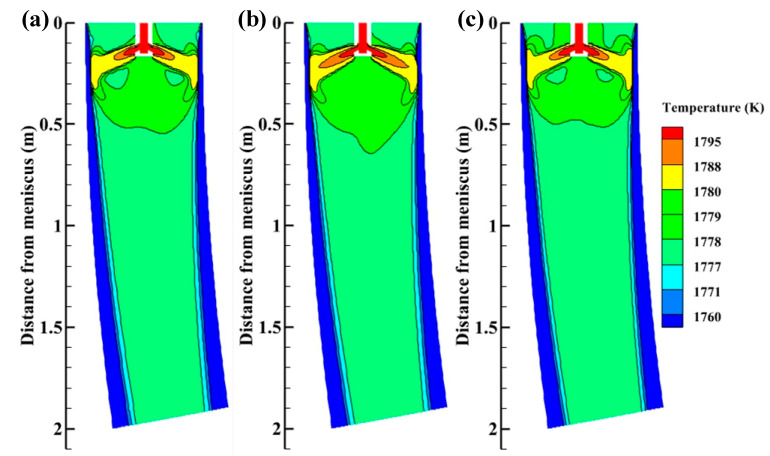
Temperature distributions on the plane Y = 0 m without M-EMS (**a**) –15° SFN, (**b**) 0° SEN, and (**c**) +15° SFN.

The temperature distributions on the plane Y = 0 m without M-EMS are presented in Figure 9. The high-temperature zone of molten steel is located in the high-speed jet zone of the nozzle, and the temperature decreases rapidly along the jet direction. SFN can increase the molten steel temperature at the meniscus, which is beneficial to the melting of mold flux and the formation of slag film in the mold, [27] reducing the possibility of casting cracks and steel breakout. Due to the sufficient stirring of the steel by the SFN, the two low-temperature zones are created by the low-temperature steel reflux. SFN makes the isotherm move up, improves mold cooling efficiency, and realizes low-temperature casting. Low-temperature casting can increase equiaxed crystal rate, reduce central segregation and improve the internal quality of round bloom.

**Figure 10:**
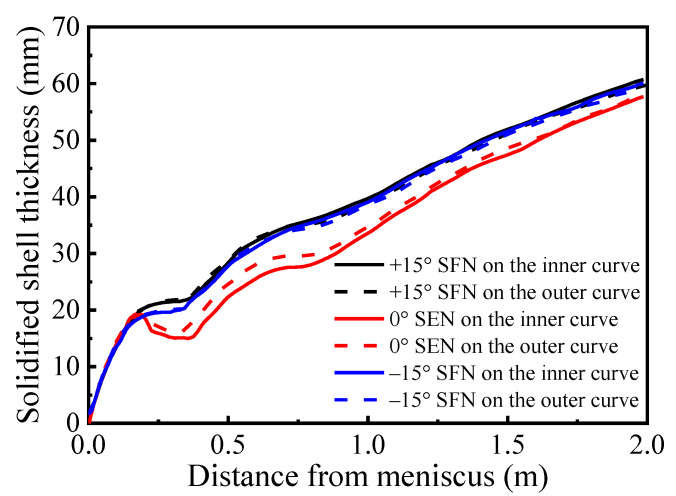
Shell thickness distributions along casting speed direction without M-EMS.

Shell thickness distributions along the casting speed direction on the Y = 0 m plane without M-EMS are shown in Figure 10. The shell thickness is considered as the distance between the billet surface and the location at 0.3 of liquid fraction, according to Aboutalebi et al. [28]. In the range of 0~0.16 m from the meniscus, the growth of the solidified shell for different nozzle structures is basically the same. In the range of 0.16~0.35 m from the meniscus, solidified shell thickness of 0° SEN shows a decreasing trend with increasing distance from the meniscus. This phenomenon is caused by the molten steel from the high-speed nozzle jet directly washing the solidified shell, resulting in a reduction and uneven distribution of the solidified shell thickness, which is consistent with the research results of Zhang [29,30] and Li [31]. With the increase in the distance from the meniscus, the solidified shell of ±15° SFN still shows an upward trend, which basically eliminates the “washing effect” of the original SEN, which is beneficial to the stable growth of the initial shell and reduces the possibility of cracks in the continuous casting process.

The shell thicknesses of 0° SEN in the inner and outer curves at the mold outlet are 27.48 mm and 29.53 mm, respectively. The thickness of the solidified shell on the inner curved side is smaller than that on the outer curved side, and the difference at the mold outlet is 2.05 mm. The solidified shell thickness on the inner curved side at the mold outlet is less than that on the outer curved side, with a difference of 2.05 mm. This is mainly due to the asymmetry of the flow field between the inner and outer curves of the mold caused by the mold curvature. According to Figure 6, compared with the outer curved side, the solidified shell on the inner curved side of the mold is more severely washed by high-temperature molten steel, the impact depth is larger, and the growth rate of the solidified shell is slower than that of the outer curve. The solidified shell thicknesses of +15° SFN in the inner and outer curves at the mold outlet are 34.48 mm and 34.47 mm, respectively. The solidified shell thickness in the inner and outer curves at the mold outlet are 33.77 mm and 33.75 mm, respectively. The solidified shell thickness distribution of ±15° SFN on the inner and outer curves is basically the same. The horizontal swirl produced by the molten steel through the swirl nozzle significantly weakens the washing of the high-temperature molten steel on the initial shell and eliminates the uneven growth of the solidified shell caused by the mold curvature. SFN is beneficial to the stable growth of solidified shells in the mold and improves mold cooling efficiency.

### 3.3. Effect of M-EMS on the Steel Flow and Heat Transfer

#### 3.3.1. Effect of M-EMS on the Steel Fluid Flow

**Figure 11:**
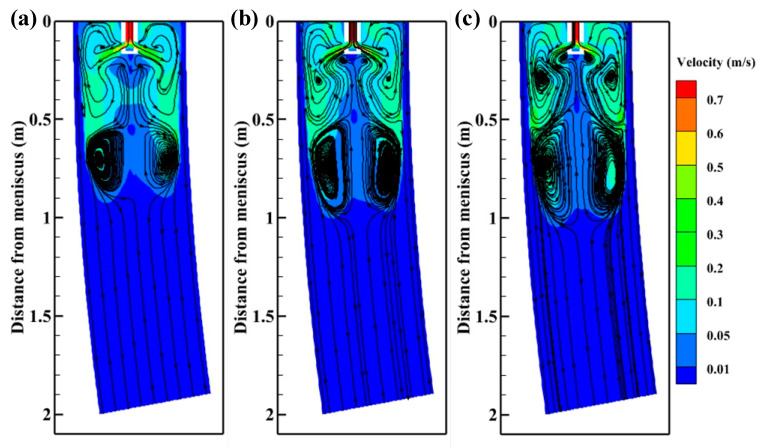
Velocity contours and streamlines on the plane Y = 0 m with M-EMS (**a**) –15° SFN, (**b**) 0° SEN, and (**c**) +15° SFN.

Figure 11 displays the velocity contours and streamlines on the plane Y = 0 m with M-EMS. With the application of M-EMS, it can be observed that the steel streamlines have changed significantly for different nozzle structures. Due to the horizontal tangential velocity of the molten steel, the upper recirculation zone is significantly lengthened along the casting direction, and a larger lower recirculation zone is formed near the mold outlet. At the same time, the third small recirculation zone formed by ±15° SFN near the meniscus disappears due to the M-EMS. Because the tangential direction of –15° SFN is opposite to the M-EMS direction, the streamlines of molten steel in the upper recirculation zone are relatively sparse. The tangential direction of +15° SFN is consistent with the M-EMS direction, the streamlines of molten steel in the upper recirculation zone become dense, and the lower recirculation zone of molten steel is relatively longer. The streamlines density of the 0° SEN is between the two kinds of SFNs.

**Figure 12:**
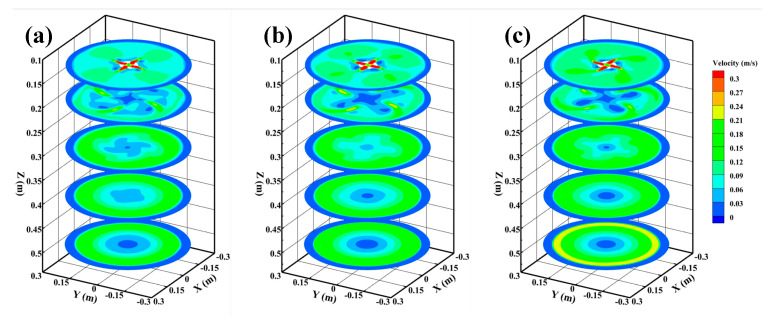
Velocity distributions in cross section of strand with M-EMS (**a**) –15° SFN, (**b**) 0° SEN, and (**c**) +15° SFN.

Figure 12 shows the velocity distributions in cross section of the strand with M-EMS, which are Z = 0.13 m, Z = 0.2 m, Z = 0.3 m, Z = 0.4 m, and Z = 0.5 m planes flow field velocity distribution, respectively. After the application of M-EMS, the tangential velocity of molten steel is higher. After the molten steel on the Z = 0.13 m plane flows from the nozzle with four high-speed jets, it is rapidly dispersed by M-EMS, which significantly reduces the washing of the high-speed steel on the initial solidified shell. The steel jets of –15° SFN show turbulent characteristics on the Z = 0.2 m plane, which is caused by the opposite direction of –15° SFN tangential direction and the M-EMS direction. The steel of +15° SFN shows an overall rotation towards the right. When the steel continues to flow to Z = 0.3 m, Z = 0.4 m, and Z = 0.5 m planes, the steel flow phenomenon at different nozzles tends to be the same, with the maximum flow rate of +15° SFN, the middle flow rate of 0° SEN, and the minimum flow rate of –15° SFN steel.

**Figure 13:**
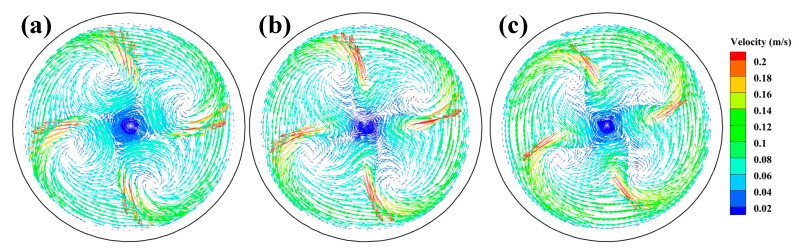
Velocity vector on the plane Z = 0.2 m with M-EMS (**a**) –15° SFN, (**b**) 0° SEN, and (**c**) +15° SFN.

Figure 13 presents the velocity vector distributions on the plane Z = 0.2 m with M-EMS. After the application of M-EMS, the steel generates a significant tangential velocity, and the steel flow rate at the solidification front is significantly reduced due to the wall effect. The tangential velocity of molten steel can prevent the growth of columnar crystals, promote the growth of equiaxed crystals, and improve mold cooling efficiency [32]. The molten steel of –15° SFN generates four obvious vortexes, indicating the presence of some turbulence in this zone. The vortex phenomenon of +15° SFN steel becomes weaker, and the tangential velocity is larger, which is beneficial to give full effect to the M-EMS.

#### 3.3.2. Effect of M-EMS on Heat Transfer and Solidification

**Figure 14:**
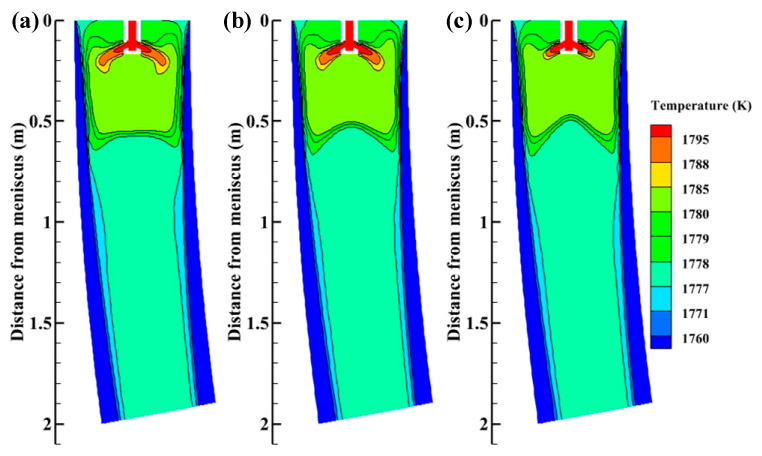
Temperature distributions on the plane Y = 0 m with M-EMS (**a**) –15° SFN, (**b**) 0° SEN, and (**c**) +15° SFN.

Figure 14 depicts the temperature distributions on the plane Y = 0 m with M-EMS. When M-EMS is turned on, the high-temperature zone in the upper part of the mold is enlarged, and the temperature near the meniscus is significantly increased compared with that without M-EMS, which is beneficial to the melting of the meniscus flux [27]. Since the mold is the greatest cooling intensity zone in the continuous casting process, if more heat is left in the mold, it is beneficial to the rapid release of superheat and improves mold cooling efficiency. This achieves the same effect as low-superheat casting, which can effectively increase equiaxed crystal rate, reduce central segregation and improve the internal quality of round bloom [33,34]. The high-temperature jet zone of +15° SFN is shorter than other nozzle structures. Because the swirl direction of +15° SFN is the same as that of M-EMS, the tangential velocity of molten steel from +15° SFN is higher, the high-temperature jet is dispersed at a faster speed, and the high-temperature jet zone becomes shorter. The length of the high-temperature jet zone from 0° SEN is in the middle. The high-temperature jet region of –15° SFN is the longest. The reason is that the swirl direction of the –15° SFN is opposite to the direction of M-EMS, which corresponds to the flow field in Figure 11.

**Figure 15:**
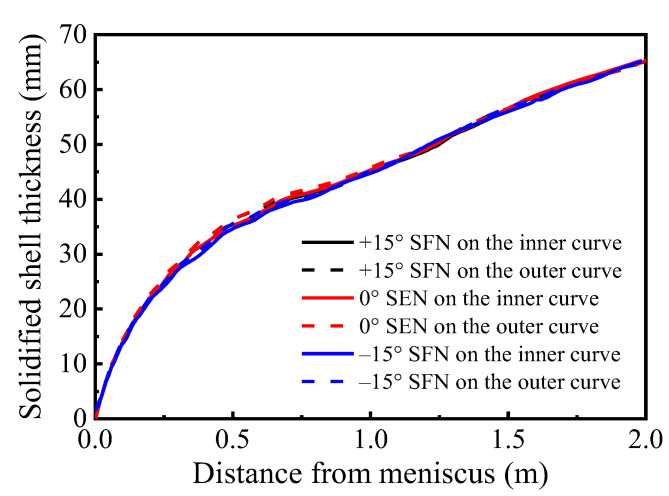
Shell thickness distributions along casting speed direction on the plane Y = 0 m with M-EMS.

Figure 15 presents solidified shell thickness distributions along the casting speed direction on the plane Y = 0 m with M-EMS. When M-EMS is on, the solidified shell thickness for different nozzle structures is similar, and it shows an increasing trend with increasing distance from the meniscus. With the application of EMS, the solidified shell thickness does not decrease or stagnate at a distance of 0.16 to 0.35 m from the meniscus. The horizontal swirl produced by M-EMS eliminates the washing effect caused by the nozzle. In the mold outlet and secondary cooling zone, the solidified shell with M-EMS is thicker than that without M-EMS. This is different from the research results of some scholars [35,36], who affirmed that the washing effect produced by M-EMS would lead to slow growth or even stagnation of solidified shells. However, M-EMS can improve heat transfer efficiency and accelerate the heat release of molten steel. M-EMS can play a good cooling role. In this paper, the reduction in solidified shell thickness caused by the washing effect of M-EMS is smaller than the increase in shell thickness caused by improving heat transfer efficiency and prolonging the steel residence time in the mold. At the same time, M-EMS eliminates the washing effect caused by the nozzle; therefore, the application of M-EMS is more favorable to the growth of shells.

### 3.4. Effect of Nozzle Structure on Level Fluctuation

**Figure 16:**
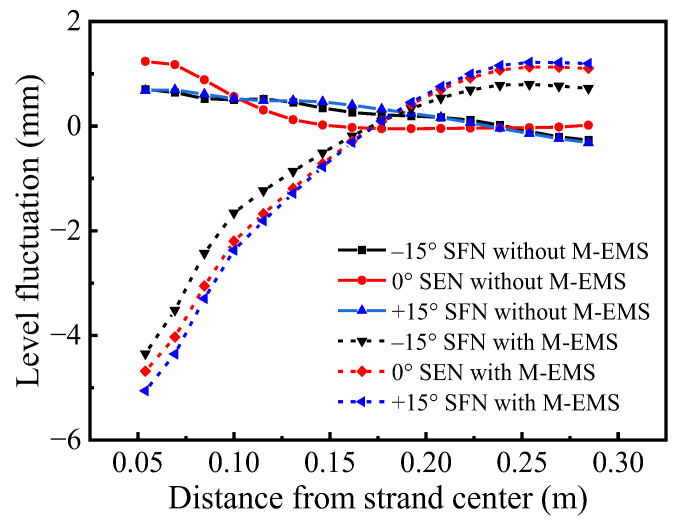
Level fluctuations along the centerline of the meniscus.

Figure 16 shows the level fluctuations along the centerline of the meniscus. The level fluctuation of the meniscus can be expressed by the relationship between the pressure at any point and the average pressure [37]. When the M-EMS is off, the overall liquid level fluctuates more smoothly. The jet of 0° SEN impinges directly on the initial solidified shell, causing large level fluctuations, which is 1.29 mm. Due to the existence of a horizontal swirl of –15° SFN and +15° SFN, the liquid level fluctuation in the meniscus is reduced, and the level fluctuation is 0.97 mm and 1.01 mm, respectively. With the application of M-EMS, the meniscus is parabolic with a low center and high edge. The geometric tangential direction of –15° SFN is opposite to that of M-EMS, which weakens the influence of M-EMS on level fluctuation, and the liquid level fluctuation is lower at 5.07 mm. The geometric tangential direction of +15° SFN is the same as that of M-EMS, aggravating the liquid level fluctuation of the meniscus, which is the largest at 6.29 mm. The 0° SEN is between the two kinds of SFNs, and the liquid level fluctuation is 5.81 mm. The predicted level fluctuation trend of the meniscus by M-EMS is consistent with the results of Liu et al. [38]. The concave liquid level fluctuation formed by the meniscus is caused by a strong swirl caused by electromagnetic stirring. The concave liquid level fluctuation near the nozzle is caused by the strong swirl of M-EMS. Compared with the unused M-EMS, the liquid level fluctuations of different nozzle structures are intensified, which is beneficial to maintain the interfacial activity, but may lead to slag entrapment [23]. The –15° SFN generates a more reasonable level of fluctuation and the lowest probability of slag entrapment, which is conducive to improving the surface quality of the round bloom.

**Figure 17:**
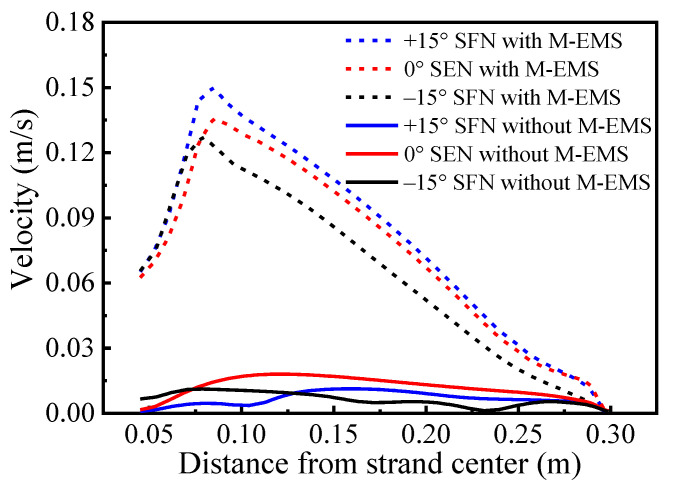
Tangential velocity distributions along the centerline of the meniscus.

The tangential velocity distributions along the centerline of the meniscus as shown in Figure 17. When M-EMS is off, the maximum tangential velocities of –15° SFN, 0° SEN, and +15° SFN are smaller, which are 0.011 m/s, 0.018 m/s, and 0.011 m/s, respectively. The tangential velocity of ±15° SFN fluctuates on the meniscus, which represents the vortex phenomenon of molten steel in the meniscus. When M-EMS is turned on, the maximum tangential velocities of –15° SFN, 0° SEN, and +15° SFN are significantly increased, which are 0.125 m/s, 0.136 m/s, and 0.150 m/s, respectively. When the meniscus velocity is too high, the periodic level fluctuation in the mold will cause exposure to the molten steel surface, resulting in the secondary oxidation of molten steel and slag entrapment. The –15° SFN should be selected if M-EMS is used. It should be avoided to use +15° SFN and M-EMS at the same time.

## 4. Conclusions

(1) Compared with the original SEN, SFN forms the third small recirculating zone near the meniscus, which can promote the flow of molten steel to the meniscus and increase the steel temperature at the meniscus. SFN can shift the isotherm upward, improve the mold cooling efficiency and realize low-temperature casting.
(2) The horizontal swirl produced by SFN reduces the washing of the high-temperature molten steel on the initial shell and eliminates the uneven growth of the solidified shell caused by mold curvature.
(3) The effect of M-EMS is similar to that of SFN. With the application of M-EMS, the high-temperature zone in the mold’s upper part enlarges, which promotes the steel superheat dissipation and is beneficial to the stable growth of the solidified shell.
(4) When the M-EMS direction is opposite to the SFN direction, the tangential velocity and the level fluctuation of the meniscus can be reduced. When the M-EMS direction is the same as the SFN direction, the level fluctuation increases, which easily produces slag entrapment.

## Figures and Tables

**Table 1:** Geometric dimensions of the three kinds of nozzles.

Parameters	SEN	SFN
Inner diameter (mm)	45	45
External diameter (mm)	90	90
Port height (mm)	35	35
Port width (mm)	25	25
Port angle (°)	0	±15
Immersion depth (mm)	100	100

**Table 2:** Chemical compositions of P91 (wt.%).

C	Si	Mn	Cr	Mo	*P*	S	Fe
0.12	0.3	0.38	8.19	0.37	0.01	0.007	Bal.

**Table 3:** Process conditions and physical parameters.

Parameters	Values
Round bloom dimension (mm)	Φ600
Casting machine radius (mm)	14,000
Mold length (mm)	700
Casting speed (m/min)	0.24
Current intensity of M-EMS (A)	300
Current frequency of M-EMS (Hz)	2
Viscosity (Pa·s)	0.0053
Density (kg/m^3^)	7000
Liquidus temperature (K)	1778
Solidus temperature (K)	1683
Specific heat (J/kg·K)	750
Latent heat of fusion (J/kg)	250,000

## Data Availability

Available on request.
